# *N*-[(*Z*)-3-(4-Chloro­benzo­yl)-1,3-thia­zolidin-2-yl­idene]cyanamide

**DOI:** 10.1107/S1600536808036568

**Published:** 2008-11-13

**Authors:** Jian-Gang Wang, Li-Hua Huang, Fang-Fang Jian

**Affiliations:** a Microscale Science Institute, Biology Department, Weifang University, Weifang 261061, People’s Republic of China; b New Materials and Functional Coordination Chemistry Laboratory, Qingdao University of Science and Technology, Qingdao 266042 People’s Republic of China; c Microscale Science Institute, Weifang University, Weifang 261061, People’s Republic of China

## Abstract

The title compound, C_11_H_8_ClN_3_OS, was prepared by the reaction of *N*-cyano­imino­thia­zolidine, 2-amino­ethanethiol and triethyl­amine at 350 K. The dihedral angle between the two rings is 62.5 (8)°.

## Related literature

For the biological activities of thia­zolidine compounds, see: Iwata *et al.* (1988 ▶); Huang & Shi (1990 ▶). For related structures, see Jian *et al.* (2006 ▶); Schroth *et al.* (1997 ▶).
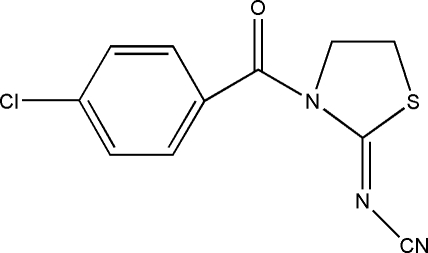


## Experimental

### 

#### Crystal data

C_11_H_8_ClN_3_OS
*M* *_r_* = 265.72
Monoclinic, 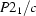

*a* = 16.442 (3) Å
*b* = 5.6798 (11) Å
*c* = 13.313 (3) Å
β = 112.76 (3)°
*V* = 1146.5 (5) Å^3^
*Z* = 4
Mo *K*α radiation
μ = 0.50 mm^−1^
*T* = 293 (2) K
0.34 × 0.21 × 0.15 mm

#### Data collection

Bruker SMART CCD area-detector diffractometer
Absorption correction: none
8343 measured reflections
2016 independent reflections
1915 reflections with *I* > 2σ(*I*)
*R*_int_ = 0.030

#### Refinement

*R*[*F* ^2^ > 2σ(*F* ^2^)] = 0.026
*wR*(*F* ^2^) = 0.066
*S* = 1.13
2016 reflections
155 parameters
H-atom parameters constrained
Δρ_max_ = 0.21 e Å^−3^
Δρ_min_ = −0.22 e Å^−3^

### 

Data collection: *SMART* (Bruker, 2001 ▶); cell refinement: *SAINT* (Bruker, 2001 ▶); data reduction: *SAINT*; program(s) used to solve structure: *SHELXS97* (Sheldrick, 2008 ▶); program(s) used to refine structure: *SHELXL97* (Sheldrick, 2008 ▶); molecular graphics: *SHELXTL* (Sheldrick, 2008 ▶); software used to prepare material for publication: *SHELXTL*.

## Supplementary Material

Crystal structure: contains datablocks global, I. DOI: 10.1107/S1600536808036568/pk2128sup1.cif

Structure factors: contains datablocks I. DOI: 10.1107/S1600536808036568/pk2128Isup2.hkl

Additional supplementary materials:  crystallographic information; 3D view; checkCIF report

## Figures and Tables

**Fig. 1.**
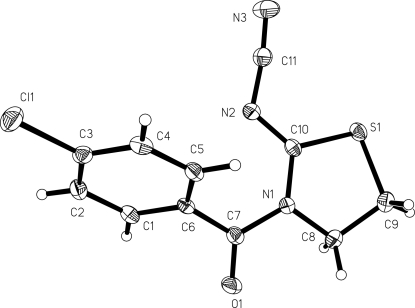
The molecular structure of the title compound with the atom-labeling scheme. Displacement ellipsoids are drawn at the 40% probability level.

## supplementary crystallographic information

### Comment

Thiazolidine is an important group in organic chemistry. Many compounds containing thiazolidine groups possess a broad spectrum of biological activities (Iwata *et al.*, 1988; Huang & Shi, 1990).

In the crystal structure (Fig. 1), the torsion angle formed by atoms N1, C8, C9 and S1 was 34.5 (9)°. The dihedral angle formed by the the ring (N1, C8, C9, C10 and S1) and the phenyl ring (C1-C6) was 62.5 (8)°. The C=N bond length (1.299 (2) Å) is in agreement with that observed before (Jian *et al.*, 2006). The C—S bond length (1.734 (7) and 1.808 (2) Å) are in agreement with those observed before (Schroth *et al.*, 1997). Intermolecular C–H···N interactions help to stabilize the crystal structure.

### Experimental

A mixture of *N*-cyanoiminothiazolidine 10 mmol (1.27 g), 2-amino-ethanethiol (1.75 g, 10 mmol) and (1.01 g, 10 mmol) triethylamine was refluxed in absolute acetone (25 ml) for 4 h. On cooling, the product crystallized, was filtered, and recrystallized from absolute EtOH (yield 2.42 g (91%)). Single crystals suitable for X-ray measurements were obtained by recrystallization from ethanol at room temperature.

### Refinement

H atoms were positioned geometrically and allowed to ride on their parent atoms, with C—H distances of 0.93–0.97 Å, respectively, and *U*_iso_(H) = 1.2 *U*_eq_ of the parent atoms.

### Crystal data

**Table:**

C_11_H_8_ClN_3_OS	*F*_000_ = 544
*M**_r_* = 265.72	*D*_x_ = 1.539 Mg m^−^^3^
Monoclinic, *P*2_1_/*c*	Mo *K*α radiation λ = 0.71073 Å
Hall symbol: -P 2ybc	Cell parameters from 1021 reflections
*a* = 16.442 (3) Å	θ = 2.9–26.4º
*b* = 5.6798 (11) Å	µ = 0.50 mm^−^^1^
*c* = 13.313 (3) Å	*T* = 293 (2) K
β = 112.76 (3)º	Block, colorless
*V* = 1146.5 (5) Å^3^	0.34 × 0.21 × 0.15 mm
*Z* = 4	

### Data collection

**Table:**

Bruker SMART CCD area-detector diffractometer	1915 reflections with *I* > 2σ(*I*)
Radiation source: fine-focus sealed tube	*R*_int_ = 0.030
Monochromator: graphite	θ_max_ = 25.0º
*T* = 293(2) K	θ_min_ = 3.1º
φ and ω scans	*h* = −19→19
Absorption correction: none	*k* = −6→6
8343 measured reflections	*l* = −15→15
2016 independent reflections	

### Refinement

**Table:**

Refinement on *F*^2^	Hydrogen site location: inferred from neighbouring sites
Least-squares matrix: full	H-atom parameters constrained
*R*[*F*^2^ > 2σ(*F*^2^)] = 0.026	*w* = 1/[σ^2^(*F*_o_^2^) + (0.017*P*)^2^ + 0.7287*P*] where *P* = (*F*_o_^2^ + 2*F*_c_^2^)/3
*wR*(*F*^2^) = 0.066	(Δ/σ)_max_ < 0.001
*S* = 1.13	Δρ_max_ = 0.21 e Å^−^^3^
2016 reflections	Δρ_min_ = −0.22 e Å^−^^3^
155 parameters	Extinction correction: SHELXL97 (Sheldrick, 2008), Fc^*^=kFc[1+0.001xFc^2^λ^3^/sin(2θ)]^-1/4^
Primary atom site location: structure-invariant direct methods	Extinction coefficient: 0.031 (2)
Secondary atom site location: difference Fourier map	

### Special details

**Table:**

Geometry. All e.s.d.'s (except the e.s.d. in the dihedral angle between two l.s. planes) are estimated using the full covariance matrix. The cell e.s.d.'s are taken into account individually in the estimation of e.s.d.'s in distances, angles and torsion angles; correlations between e.s.d.'s in cell parameters are only used when they are defined by crystal symmetry. An approximate (isotropic) treatment of cell e.s.d.'s is used for estimating e.s.d.'s involving l.s. planes.
Refinement. Refinement of *F*^2^ against ALL reflections. The weighted *R*-factor *wR* and goodness of fit *S* are based on *F*^2^, conventional *R*-factors *R* are based on *F*, with *F* set to zero for negative *F*^2^. The threshold expression of *F*^2^ > σ(*F*^2^) is used only for calculating *R*-factors(gt) *etc*. and is not relevant to the choice of reflections for refinement. *R*-factors based on *F*^2^ are statistically about twice as large as those based on *F*, and *R*- factors based on ALL data will be even larger.

### Fractional atomic coordinates and isotropic or equivalent isotropic displacement parameters (Å^2^)

**Table:**

	*x*	*y*	*z*	*U*_iso_*/*U*_eq_	
S1	0.07965 (3)	0.52934 (7)	0.33331 (3)	0.02469 (14)	
Cl1	0.46689 (3)	0.71315 (8)	0.06338 (3)	0.03512 (15)	
O1	0.27367 (8)	1.17756 (19)	0.37317 (10)	0.0302 (3)	
N2	0.16368 (9)	0.5912 (2)	0.19639 (11)	0.0244 (3)	
C3	0.40564 (10)	0.7898 (3)	0.13961 (12)	0.0229 (3)	
N1	0.20390 (8)	0.8283 (2)	0.35254 (10)	0.0216 (3)	
C10	0.15500 (10)	0.6503 (3)	0.28595 (12)	0.0201 (3)	
C4	0.40148 (10)	0.6326 (3)	0.21690 (12)	0.0232 (3)	
H4A	0.4315	0.4901	0.2281	0.028*	
N3	0.08333 (10)	0.2509 (3)	0.08619 (14)	0.0426 (4)	
C7	0.26182 (10)	0.9843 (3)	0.33103 (12)	0.0220 (3)	
C6	0.30963 (9)	0.9060 (3)	0.26219 (12)	0.0197 (3)	
C8	0.16984 (11)	0.9017 (3)	0.43566 (13)	0.0263 (4)	
H8A	0.1270	1.0272	0.4077	0.032*	
H8B	0.2176	0.9574	0.5007	0.032*	
C1	0.31667 (10)	1.0641 (3)	0.18625 (13)	0.0227 (3)	
H1A	0.2896	1.2106	0.1779	0.027*	
C9	0.12728 (11)	0.6865 (3)	0.46101 (13)	0.0268 (4)	
H9A	0.0820	0.7312	0.4872	0.032*	
H9B	0.1708	0.5901	0.5158	0.032*	
C11	0.11795 (11)	0.4075 (3)	0.14112 (14)	0.0275 (4)	
C5	0.35225 (10)	0.6895 (3)	0.27740 (12)	0.0222 (3)	
H5A	0.3477	0.5833	0.3281	0.027*	
C2	0.36370 (10)	1.0057 (3)	0.12284 (13)	0.0244 (3)	
H2B	0.3669	1.1093	0.0704	0.029*	

### Atomic displacement parameters (Å^2^)

**Table:**

	*U*^11^	*U*^22^	*U*^33^	*U*^12^	*U*^13^	*U*^23^
S1	0.0224 (2)	0.0236 (2)	0.0314 (2)	−0.00304 (16)	0.01415 (17)	0.00002 (16)
Cl1	0.0368 (3)	0.0412 (3)	0.0340 (2)	0.00200 (19)	0.02096 (19)	−0.00607 (19)
O1	0.0369 (7)	0.0210 (6)	0.0374 (7)	−0.0065 (5)	0.0194 (5)	−0.0072 (5)
N2	0.0252 (7)	0.0244 (7)	0.0252 (7)	−0.0060 (6)	0.0115 (6)	−0.0039 (6)
C3	0.0187 (7)	0.0266 (8)	0.0226 (8)	−0.0038 (6)	0.0071 (6)	−0.0060 (6)
N1	0.0246 (7)	0.0207 (7)	0.0217 (6)	−0.0036 (5)	0.0114 (5)	−0.0012 (5)
C10	0.0185 (7)	0.0167 (7)	0.0243 (8)	0.0018 (6)	0.0075 (6)	0.0039 (6)
C4	0.0194 (8)	0.0190 (7)	0.0273 (8)	0.0006 (6)	0.0047 (6)	−0.0016 (6)
N3	0.0337 (8)	0.0427 (10)	0.0586 (11)	−0.0120 (8)	0.0257 (8)	−0.0256 (9)
C7	0.0218 (8)	0.0201 (8)	0.0229 (8)	−0.0012 (6)	0.0072 (6)	0.0024 (6)
C6	0.0171 (7)	0.0187 (7)	0.0219 (7)	−0.0047 (6)	0.0062 (6)	−0.0019 (6)
C8	0.0293 (8)	0.0284 (8)	0.0244 (8)	−0.0013 (7)	0.0140 (7)	−0.0026 (7)
C1	0.0209 (8)	0.0171 (7)	0.0289 (8)	−0.0013 (6)	0.0082 (6)	0.0017 (6)
C9	0.0248 (8)	0.0326 (9)	0.0236 (8)	−0.0002 (7)	0.0101 (7)	0.0028 (7)
C11	0.0230 (8)	0.0303 (9)	0.0348 (9)	−0.0025 (7)	0.0172 (7)	−0.0056 (8)
C5	0.0226 (8)	0.0192 (8)	0.0227 (8)	−0.0029 (6)	0.0064 (6)	0.0022 (6)
C2	0.0242 (8)	0.0248 (8)	0.0245 (8)	−0.0030 (7)	0.0099 (6)	0.0040 (7)

### Geometric parameters (Å, °)

**Table:**

S1—C10	1.7347 (15)	N3—C11	1.152 (2)
S1—C9	1.8082 (17)	C7—C6	1.488 (2)
Cl1—C3	1.7390 (16)	C6—C1	1.390 (2)
O1—C7	1.2136 (19)	C6—C5	1.391 (2)
N2—C10	1.299 (2)	C8—C9	1.510 (2)
N2—C11	1.329 (2)	C8—H8A	0.9700
C3—C2	1.382 (2)	C8—H8B	0.9700
C3—C4	1.384 (2)	C1—C2	1.388 (2)
N1—C10	1.379 (2)	C1—H1A	0.9300
N1—C7	1.409 (2)	C9—H9A	0.9700
N1—C8	1.4804 (19)	C9—H9B	0.9700
C4—C5	1.383 (2)	C5—H5A	0.9300
C4—H4A	0.9300	C2—H2B	0.9300
			
C10—S1—C9	92.05 (8)	N1—C8—H8A	110.5
C10—N2—C11	118.14 (14)	C9—C8—H8A	110.5
C2—C3—C4	121.83 (14)	N1—C8—H8B	110.5
C2—C3—Cl1	119.55 (12)	C9—C8—H8B	110.5
C4—C3—Cl1	118.61 (12)	H8A—C8—H8B	108.7
C10—N1—C7	127.01 (13)	C2—C1—C6	120.72 (14)
C10—N1—C8	113.06 (12)	C2—C1—H1A	119.6
C7—N1—C8	117.11 (13)	C6—C1—H1A	119.6
N2—C10—N1	122.40 (14)	C8—C9—S1	105.01 (11)
N2—C10—S1	125.37 (12)	C8—C9—H9A	110.7
N1—C10—S1	112.17 (11)	S1—C9—H9A	110.7
C5—C4—C3	119.40 (14)	C8—C9—H9B	110.7
C5—C4—H4A	120.3	S1—C9—H9B	110.7
C3—C4—H4A	120.3	H9A—C9—H9B	108.8
O1—C7—N1	118.36 (14)	N3—C11—N2	172.67 (18)
O1—C7—C6	121.78 (14)	C4—C5—C6	119.80 (14)
N1—C7—C6	119.80 (13)	C4—C5—H5A	120.1
C1—C6—C5	119.87 (14)	C6—C5—H5A	120.1
C1—C6—C7	117.87 (14)	C3—C2—C1	118.32 (14)
C5—C6—C7	122.08 (14)	C3—C2—H2B	120.8
N1—C8—C9	106.32 (13)	C1—C2—H2B	120.8
			
C11—N2—C10—N1	175.68 (14)	N1—C7—C6—C1	138.53 (15)
C11—N2—C10—S1	−7.2 (2)	O1—C7—C6—C5	130.83 (17)
C7—N1—C10—N2	6.7 (2)	N1—C7—C6—C5	−46.3 (2)
C8—N1—C10—N2	167.01 (14)	C10—N1—C8—C9	29.96 (17)
C7—N1—C10—S1	−170.72 (12)	C7—N1—C8—C9	−167.65 (13)
C8—N1—C10—S1	−10.44 (16)	C5—C6—C1—C2	1.9 (2)
C9—S1—C10—N2	172.92 (14)	C7—C6—C1—C2	177.18 (14)
C9—S1—C10—N1	−9.73 (12)	N1—C8—C9—S1	−34.60 (15)
C2—C3—C4—C5	1.7 (2)	C10—S1—C9—C8	25.81 (12)
Cl1—C3—C4—C5	−179.26 (11)	C3—C4—C5—C6	−1.8 (2)
C10—N1—C7—O1	152.51 (15)	C1—C6—C5—C4	0.0 (2)
C8—N1—C7—O1	−7.1 (2)	C7—C6—C5—C4	−175.00 (14)
C10—N1—C7—C6	−30.2 (2)	C4—C3—C2—C1	0.2 (2)
C8—N1—C7—C6	170.18 (13)	Cl1—C3—C2—C1	−178.80 (12)
O1—C7—C6—C1	−44.3 (2)	C6—C1—C2—C3	−2.1 (2)
